# Supplementary material for: Identification of Genes Associated with Gut Microbiota Metabolites in Type 2 Diabetes Mellitus Complicated by Acute Cerebral Infarction Based on Bioinformatics and Mendelian Randomization
Source: Biomedicines. 2026 Oct 17;14(10):2371. doi: 10.3390/biomedicines14102371 (PMC13702449; doi:10.3390/biomedicines14102371)
Supplement: Supplementary file 1 [file biomedicines-14-02371-s001.zip › Figure S2/genus.Sellimonas.id.14369 ACI.pdf]

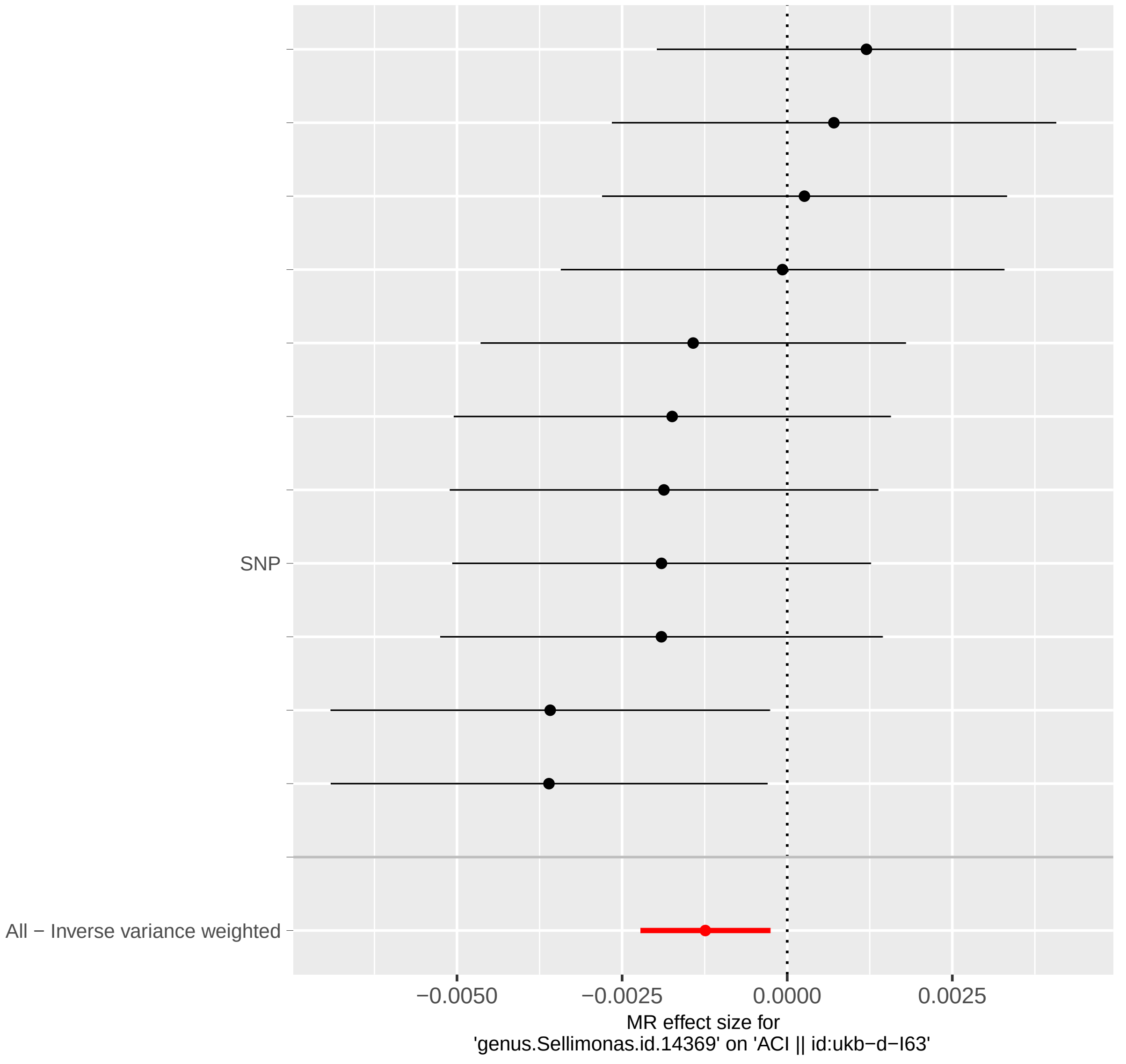
SNP
All − Inverse variance weighted
−0.0050
−0.0025
0.0000
0.0025
MR effect size for
'genus.Sellimonas.id.14369' on 'ACI || id:ukb−d−I63'
